# LBP rs2232618 polymorphism contributes to risk of sepsis after trauma

**DOI:** 10.1186/s13017-018-0214-1

**Published:** 2018-11-16

**Authors:** Hong-xiang Lu, Jian-hui Sun, Da-lin Wen, Juan Du, Ling Zeng, An-qiang Zhang, Jian-xin Jiang

**Affiliations:** State Key Laboratory of Trauma, Burns and Combined Injury, Institute of Surgery Research, Daping Hospital, Third Military Medical University, Chongqing, 400042 China

**Keywords:** Trauma, Sepsis, Lipopolysaccharide-binding protein, Single nucleotide polymorphism, Meta-analysis

## Abstract

**Background:**

Previous study revealed that rs2232618 polymorphism (Phe436Leu) within LBP gene is a functional variant and associated with susceptibility of sepsis in traumatic patients. Our aim was to confirm the reported association by enlarging the population sample size and perform a meta-analysis to find additional evidence.

**Methods:**

Traumatic patients from Southwest (*n* = 1296) and Southeast (*n* = 445) of China were enrolled in our study. After genotyping, the relationship between rs2232618 and the risk of sepsis was analyzed. Furthermore, we proceeded with a comprehensive literature search and meta-analysis to determine whether the rs2232618 polymorphism conferred susceptibility to sepsis.

**Results:**

Significance correlation was observed between rs2232618 and risk of sepsis in Southwest patients (*P* = 0.002 for the dominant model, *P* = 0.006 for the recessive model). The association was confirmed in Southeast cohort (*P* = 0.005 for the dominant model) and overall combined cohorts (*P* = 4.5 × 10^−4^, *P* = 0.041 for the dominant and recessive model). Multiple logistical regression analyses suggested that rs2232618 polymorphism was related to higher risk of sepsis (OR = 1.77, 95% CI = 1.26–2.48, *P* = 0.001 in Southwest patients; OR = 2.11, 95% CI = 1.24–3.58, *P* = 0.006 in Southeast cohort; OR = 1.54, 95% CI = 1.34–2.08, *P* = 0.006 in overall cohort). Furthermore, meta-analysis of four studies (including the present study) confirmed that rs2232618 within LBP increased the risk of sepsis (OR = 1.75, *P* < 0.001 for the dominant model; OR = 6.08, *P* = 0.003 for the recessive model; OR = 2.72, *P* < 0.001 for the allelic model).

**Conclusions:**

The results from our replication study and meta-analysis provided firm evidence that rs2232618T allele significantly increased the risk of sepsis.

## Backgrounds

According to WHO, 10% of deaths and 16% of disabilities around the world were due to traumatic injuries [1]. With the development of first aid and hospital treatment, the early mortality of major trauma patients declined in recent years [2]. However, the incidence of mortality caused by post-injury sepsis remained unchanged during the past decades [3, 4]. Despite the obtained increasing research progress in sepsis after trauma, current knowledge about the molecular mechanisms of the development of sepsis is still limited [5]. Therefore, early diagnosis and treatment based on the special clinical signs and laboratory results become imperative requirements [6].

Previous studies indicated that gene variants (generally single nucleotide polymorphisms, SNPs) in inflammatory response genes could contribute to different outcomes which are observed in sepsis and infectious diseases both in laboratory animal models and clinical patient cohorts [7, 8]. Candidate gene studies for traumatic patients identified several SNPs in lipopolysaccharide-binding protein (LBP), toll-like receptor 1(TLR1), and tumor necrosis factor-alpha (TNF-α) which were related to the development of sepsis [9–11]. The assessment of sepsis-specific genetic variants in these patients could explain the individual differences in susceptibility for trauma-related sepsis to some extent [7, 12]. Therefore, those SNPs could serve as beneficial biomarkers to evaluate and monitor infection or inflammatory responses to trauma patients.

Lipopolysaccharide-binding protein (LBP), a key gene in the host innate immune response, has been reported to play a crucial role in the pathophysiologic process of sepsis after major traumatic injury [13]. We previously found that the rs2232618 (Phe436Leu) polymorphism in LBP had a significant association with the incidence of sepsis and MOD score in two non-dependent cohorts of major traumatic patients admitted from Chongqing (Southwest of China) and Zhejiang (Southeast of China). The correlation analysis showed these patients with variant C allele had higher sepsis morbidity risk and MOD score. Other studies also showed that rs2232618 could affect the outcome of sepsis patients [14, 15]. In addition, protein activities could enhance after C allele mutated to T allele at rs2232618 [16]. Thus, the current study was designed to examine the association between rs2232618 and sepsis after trauma by enlarging the sample size. Furthermore, a meta-analysis including previously published studies was carried out to provide a more precise estimate of this association.

## Materials and methods

### Study populations

Two unrelated study cohorts of traumatic injury patients in Southwest (Chongqing) and Southeast (Zhejiang) of China were performed for this study. Traumatic patients in the ICU at the Department of Trauma Surgery in the Daping Hospital and the Chongqing Emergency Medical Center were recruited during the period of between January 2005 and October 2016. The traumatic injury patients in the Second Affiliated Hospital, Zhejiang University, were enrolled between January 2008 and July 2015. The including criteria and excluding criteria were described previously [16]. Trauma severity of each person was assessed using the Injury Severity Score (ISS) (The Abbreviated Injury Scale: 2005 revisions) by two independent researchers. Demographic characteristics and clinical information were taken from the electronic medical record. Consequently, the diagnosis of sepsis was according to the criteria of the American College of Chest Physicians and Society of Critical Care Medicine Consensus Committee. Definition of infection was clinically positive bacterial cultures from blood, sputum, urine tissue, catheter tips, and wounds. For those trauma patients with multiple positive cultures, the first significant culture of gram-positive or gram-negative organisms occurring after admission was selected. Multiple organ dysfunction (MOD) score was the sum of single organ score calculated during every day the patients stayed in the hospital. The patient sampling and experiments got approval from the Institutional Ethics Review Board of the Third Military Medical University. Informed consent for all subjects was acquired from the patients or their kin.

### Genotyping

Blood samples of trauma patients were obtained immediately after admission by physicians or nurses. Total DNA of every patient was extracted from whole blood according to the laboratory protocol. Samples were stored at − 80 °C with a 40 μg/ml concentration. Pyrosequencing was utilized to genotype rs2232618 similar to our previous report [16, 17]. The double-blind method was implemented. Approximately 10% of the samples was genotyped in duplicate to ensure genotyping quality.

### Statistical analysis

Categorical data were shown as counts and percentages. Continuous data were given as means ± SD. Comparison of categorical data was conducted by *χ*^2^ analysis, and continuous data were analyzed using Student’s *t* test. Genotype frequencies were determined according to gene number. Hardy-Weinberg equilibrium (HWE) was assessed to detect whether the rs2232618 polymorphism distribution among the study population was stable by *χ*^2^ analyses. The correlation between rs2232618 polymorphisms and the incidence of sepsis was performed by *χ*^2^ analyses in three genetic effects (allele dose genetic model, dominant genetic model, and recessive genetic model). Furthermore, the allelic odds ratio (OR) and 95% confidence intervals (CI) were calculated by a multiple stepwise logistic regression analysis adjusted by identified confounding variables of age, sex, and ISS. Moreover, we also compared the MOD scores between different genotypes with Student’s *t* test. The exact *P* values were considered significant if *P* < 0.05. All statistical analyses were performed in SPSS 17.

### Meta-analysis of rs2232618 in association with sepsis risk

To confirm the involvement of rs2232618 in sepsis susceptibility, a meta-analysis combining published studies and our study was carried out. PubMed, Embase, and Web of Knowledge were searched in order to identify all published studies up to December 15, 2017, that had evaluated the associations between rs2232618 polymorphism and sepsis. Key words used for search were “rs2232618 or Leu436Pro” and “sepsis or severe sepsis or septic shock or septicemia.” The inclusion criteria were as follows: (1) independent case-control or cohort study evaluating the association between rs2232618 and sepsis risk and (2) the number or frequency of genotypes was provided in detail or obtained by contacting the authors.

Information such as first author’s name, publication year, country origin and the ethnicity of study population, genotype number, or allele frequency for case and control were collected from each study using a standardized data collection protocol. The odds ratio (OR) and its 95% confidence interval (CI) were used to evaluate the strength of the association between rs2232618 and sepsis susceptibility based on genotype frequencies in cases and controls. The pooled ORs were performed for dominant (TT versus CC + CT), recessive (TT + CT versus CC), and allelic (T versus C) genetic models, respectively. The significance of pooled ORs was tested by *Z* test (*P* < 0.05 was considered statistically significant).

Between-study heterogeneity across all eligible comparisons was estimated by the Cochran’s *Q* statistic and the *I*^2^ metric. Heterogeneity was considered significant at *P* < 0.05 for the *Q* statistic. For the *I*^2^ metric, the following cut-off points were used: *I*^2^ = 0–25%, no heterogeneity; *I*^2^ = 25–50%, moderate heterogeneity; *I*^2^ = 50–75%, large heterogeneity; *I*^2^ = 75–100%, extreme heterogeneity. A fixed-effects model, using Mantel-Haenszel method, was applied to pool data from studies when heterogeneity was negligible based on *P* for *Q* statistic greater than 0.1; otherwise, a random-effects model, using DerSimonian and Laird method was applied. The meta-analysis was conducted using Review Manager 5.0.

## Results

### Overall clinical characteristics of major traumatic patients

There were1296 major traumatic patients from Southwest of China and 445 patients from Southeast of China enrolled and genotyped in our study. The demographic and clinical information of those patients was presented in Table 1. Most of the trauma patients were male. Patients were of young age (mean age 42.5 ± 12.9 and 41.4 ± 12.3). All patients in the study survived more than 48 h after admitted to the hospital. Average ISS in Southwest and Southeast are 21.2 ± 9.4 and 21.7 ± 9.3, respectively. Among them, incidence of trauma sepsis is 33.3% and 37.5% in the Southwest and Southeast of China, respectively. The main type of infection was respiratory tract infection in the two study cohorts (27.6% and 43.1%). According to infection of bacterial species, gram-negative infections occupied about 41.4% and 38.9% and gram-positive infections were about 29.6% and 9.6%. Among the trauma population, the mean of MOD score was 7.17 ± 1.02 and 6.41 ± 0.85 in Southwest and Southeast, respectively.

**Table 1:** Overall clinical characteristics of patients with major trauma

Variables	Southwest (*n* = 1296)	Southeast (*n* = 445)
Age, years	42.5 ± 12.9	41.4 ± 12.3
Male/female, %	81.2/18.8	77.8/22.2
AIS max abdomen	2.6 ± 0.9	2.5 ± 0.6
AIS max extremities/pelvis	2.7 ± 0.8	2.8 ± 0.5
AIS max face	1.5 ± 0.7	1.7 ± 0.3
AIS max head/neck	2.9 ± 1.3	2.5 ± 1.1
AIS max thorax	3.1 ± 0.6	3.4 ± 0.2
ISS	21.2 ± 9.4	21.7 ± 9.3
MOD scores	7.17 ± 1.02	6.41 ± 0.85
Sepsis, *n* (%)	432 (33.3%)	167 (37.5%)
Source of infection, *n* (%)		
Respiratory tract infection, *n* (%)	70 (27.6)	72 (43.1)
Primary bloodstream infection, *n* (%)	43 (16.5)	33 (19.8)
Urinary tract infection, *n* (%)	24 (9.2)	20 (12.0)
Catheter associated infection, *n* (%)	55 (21.1)	15 (9.0)
Wound infection, *n* (%)	44 (16.9)	17 (10.1)
Others, *n* (%)	18 (6.8)	9 (6.0)
Pathogens, *n* (%) (positive blood cultures)		
Gram-negative, *n* (%)	179 (41.4)	65 (38.9)
Gram-positive, *n* (%)	128 (29.6)	16 (9.6)
Fungi, *n* (%)	4 (0.9)	0 (0)
Mixed gram-negative and gram-positive, *n* (%)	5 (1.2)	0 (0)
Negative blood cultures, *n* (%)	116 (26.9)	86 (51.5)

### Clinical correlation of the rs2232618with trauma-related sepsis

The rs2232618 was successfully genotyped in 1296 Southwest of China trauma patients. The overall minor allele frequency (MAF = 5.5%) was consistent with the 86 Chinese Han Beijing in HapMap datasets (MAF = 9.1%). The genotype frequencies of rs2232618 was in line with Hardy-Weinberg equilibrium (*P* = 0.06) (Table 2). Both allele and genotype frequencies of rs2232618 remained constant in the Southwest cohort. As presented in Table 3, no statistically significant difference in age, gender, or ISS was detected among traumatic patients with different genotypes. In the Southwest cohort, we found a strong association between rs2232618 and incidence of sepsis both in the dominant model (*P* = 0.002) and in recessive effect of the allele (*P* = 0.006), so the trauma patients with more C allele would be more likely to suffer from sepsis (TT 32.0%, TC 43.9%, CC 71.4%). For multiple logistical regression analyses, data from allele dose model analyses adjusted by age, sex, and ISS also suggested that rs2232618 polymorphism had a significant correlation with higher morbidity rate of sepsis (OR = 1.77, 95% CI = 1.26–2.48, *P* = 0.001) (Table 3). In addition, when comparing the MOD score among patients with different genotypes, results indicated that C carriers had a higher MOD score than the T carrier patients (*P* = 1.8 × 10^−6^ in case of dominant model) (Table 3). Therefore, C carriers may be more likely to have bad outcome.

**Table 2:** Distribution of rs2232618 in the LBP gene among trauma patients in the two cohorts

		MAF, %		Genotypes, *N*			
	Patients	Databank*	Patients	Wild	Heterozygous	Variant	HWE
Southwest	1296	9.1	5.5	1166	123	7	0.06
Southeast	445	9.1	6.1	388	54	3	0.46

*Data were from HapMap database for Chinese Han Beijing (*n* = 139)

**Table 3:** Clinical relevance of rs2232618 among trauma patients in the two cohorts

	Genotypes	*N*	Age (years)	Sex (M/F, %)	ISS	Sepsis, *n* (%)	MOD score
Southwest	TT	1166	42.6 ± 12.8	81.4/18.6	20.8 ± 9.3	373 (32.0)	6.11 ± 2.24
	TC	123	41.7 ± 13.9	77.2/22.8	25.0 ± 9.7	54 (43.9)	7.20 ± 2.23
	CC	7	43.0 ± 10.7	100/0	24.1 ± 13.2	5 (71.4)	8.17 ± 3.19
						a1, b1, c1	a2
Southeast	TT	388	41.3 ± 12.2	78.1/21.9	21.5 ± 9.3	136 (35.1)	5.88 ± 2.32
	TC	54	42.1 ± 12.9	79.6/20.4	23.4 ± 9.3	29 (53.7)	7.39 ± 3.73
	CC	3	32.7 ± 8.1	33.3/66.7	21.3 ± 7.2	2 (66.7)	6.00 ± 1.41
						a3, c2	a4
Total	TT	1554	42.3 ± 12.6	80.6/19.4	21.0 ± 9.3	509 (33.0)	6.07 ± 2.24
	TC	177	41.2 ± 13.7	92.1/7.9	22.5 ± 10.3	83 (46.9)	7.27 ± 2.87
	CC	10	43.4 ± 12.1	80.0/20.0	23.8 ± 11.9	7 (70.0)	8.00 ± 2.94
						a5, b2, c3	a6

Dominant effect (variant homozygotes + heterozygotes vs. wild homozygotes) as analyzed by ANCOVA: ^a1^*P* = 0.002, ^a2^*P* = 1.8E−6, ^a3^*P* = 0.002, ^a4^*P* = 0.005,^a5^*P* = 4.5 × 10^−4^, ^a6^*P* = 1.4E−9

Recessive effect (variant homozygotes vs. heterozygotes + wild homozygotes) as analyzed by ANCOVA: ^b1^*P* = 0.032, ^b2^*P* = 0.041

Allele dose association by logistic regression: ^c1^*P* = 0.001(OR = 1.77, 95% CI = 1.26–2.48), ^c2^*P* = 0.006(OR = 2.11, 95% CI = 1.24–3.58), ^c3^*P* = 0.005(OR = 1.54, 95% CI = 1.34–2.08)

We further validated those results in another distinct trauma cohort (Southeast of China). The characteristics and clinical data of injury patients from Southeast of China are shown in Table 1. The overall MAF of rs2232618 (MAF = 6.1%) in the validation trauma cohort was consistent with those from Southwest of China and HapMap datasets. The genotype distribution conformed to the HWE (*P* = 0.46). As shown in Table 3, the risk rate of sepsis increased when the patients were with more C allele (TT 35.1%, TC 53.75%, CC 66.7%). There was a strong association between rs2232618 and development of post-traumatic sepsis in the dominant effect (*P* = 0.005). However, relevance of rs2232618 and sepsis morbidity in recessive genetic model was not detected again; the reason might be that there were just three TT genotype trauma patients from Southeast of China and it was not enough to validate the significant association. A multiple analysis was performed by stepwise logistic regression; the results suggested that rs2232618 polymorphism was related to higher risk of sepsis (OR *=* 2.11, 95% CI = 1.24–3.58, *P =* 0.006). Furthermore, we found that the C carriers also had higher MOD score than those patients with T allele in the dominant model (*P =* 0.005) (Table 3).

Due to no significant differences in the distribution of age, sex, and injury severity among patients from Southwest and Southeast of China were identified, the two cohorts were combined to enlarge the study cohort. Just as presented in Table 3, there was a stronger relevance between rs2232618 polymorphism and incidence of sepsis or MOD scores. The results suggested that rs2232618T → C would greatly increase the risk of sepsis in dominant and recessive model (*P =* 4.5 × 10^−4^ and *P =* 0.041). Similar with previous results, allele dose effect analyses also confirmed the relevance for rs2232618 polymorphism and morbidity of sepsis (OR *=* 1.54, 95% CI = 1.34–2.08, *P =* 0.005). Furthermore, a significant difference in MOD score was observed among traumatic patients with different genotypes (*P =* 1.4×10^−9^ in dominant genetic model).

### Results of meta-analysis

**Fig. 1:**
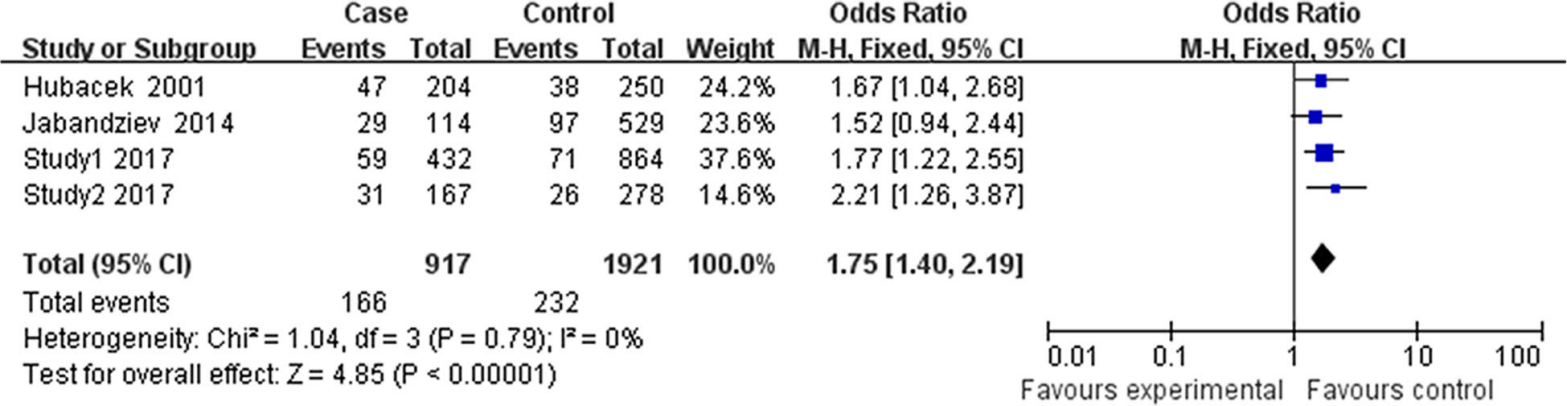
Forest plot of sepsis susceptibility associated with rs2232618 polymorphism under the dominant model (TT vs. CC + TC)

**Fig. 2:**
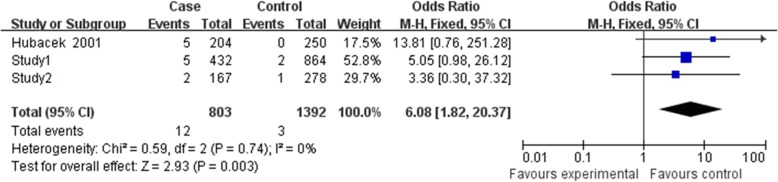
Forest plot of sepsis susceptibility associated with rs2232618 polymorphism under the recessive model (TT + CT vs. CC)

**Fig. 3:**
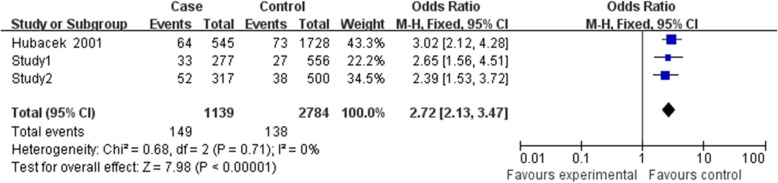
Forest plot of sepsis susceptibility associated with rs2232618 polymorphism under the allelic model (T vs. C)

Finally, three relevant articles were included in final meta-analysis [14–16]. There were 4 studies with 917 cases, and 1291 controls determined the association between rs2232618 polymorphism and sepsis risk (Table 4). However, Jabandziev’s study [15] just provided genotype number for TT vs. TT + TC, so this study was just included in the dominant genetic model. Because Zeng et al.’s Chongqing and Zhejiang cohorts were included in our study, they were presented in study 1 and study 2 [16]. As shown in Figs. 1, 2, and 3, no significant evidence of heterogeneity was observed in all genetic models (dominant model, *I*^2^ = 0, *P =* 0.79; recessive model *I*^2^ = 0, *P =* 0.74; allelic model *I*^2^ = 0, *P =* 0.71), so a fix-effects model was to pool the OR. In the dominant genetic model (TT VS. TC + CC), overall pooled OR for four studies combined was 1.75 (95% CI = 1.40–2.19) (*P* < 0.001) (Fig. 1). Similarly, the recessive and allelic models were all significantly associated with sepsis risk (recessive genetic model OR = 6.08, 95% CI = 1.82–20.37, *P* = 0.003 (Fig. 2); allelic genetic model OR = 2.72, 95% CI = 2.13–3.47, *P* < 0.001) (Fig. 3).

**Table 4:** Characteristics of the studies included in the meta-analysis

Author	Country	Ethnicity	Case/control	Case			Control		
				TT	TC	CC	TT	TC	CC
Study1^#1^	China	Han	432/864	373	54	5	793	69	2
Study2^#2^	China	Han	167/278	136	29	2	252	25	1
Jabandziev 2014*	Czech	NA	114/529	85	29		432	97	
Hubacek 2001	Germany	NA	204/250	157	42	5	212	38	0

Zeng’s Chongqing and Zhejiang cohorts were included in our study, so they were not presented independently

^#1^Study1 represented the Southwest cohorts in our study

^#2^Study2 represented Southeast cohorts in our study

*Jabandziev’s study just provided genotype number for TT vs. TT + TC. The number of TT and CC was not shown separately. 29 and 97 represented the TT + TC in case and control, respectively

## Discussion

Patients after major traumatic injury were at high risk of sepsis and sepsis-associated multiple organ dysfunction syndrome [18, 19]. Therefore, increasing interest in identifying sepsis early in clinical management and providing timely and accurate therapies shorten hospital stays and improve overall outcomes [19]. Recently, researchers paid great attention to the potential action for genetic variation in sepsis susceptibility after traumatic injury. Various investigators had detected potential relevance between immune-related gene polymorphisms and risk of septic episodes [9]. SNPs could regulate the expression of innate immune system components, inflammatory cytokines, and coagulation cascade, so illuminating the influence of variation on immune inflammatory response from a cellular and molecular level might contribute to enhance management in the later stage of trauma [15, 20]. Our study indicated that rs2232618 in LBP gene was associated with the morbidity of trauma-related sepsis and C allele carriers had higher sepsis rate in Southwest and Southwest of China trauma patients. Moreover, meta-analysis also revealed that rs2232618 was related with risk of sepsis under all genetic models.

LBP as a class I acute-phase protein of hepatic origin could mediate innate immune responses after recognizing lipopolysaccharides (LPS) originating from different gram-negative bacteria [21, 22]. LBP could form a high-affinity complex with LPS, then LPS was delivered to cell through CD14 or TLR4-MD2 and triggered a cascade of cytokines and pro-inflammatory mediators [23]. During sepsis, previous studies suggested that levels of serum LBP elevated almost seven times higher than normal levels [24]. Therefore, LBP might be a promising tool for the early clinical diagnosis of sepsis and appropriated in differentiating sepsis and systemic inflammatory response syndrome (SIRS) [25]. It was reasonable to suppose the SNP affecting the expression or activities of LBP might also have influence on individual susceptibility for sepsis. Flores et al. [26] have reported a common SNP risk haplotype of LBP gene that was strongly related to susceptibility to severe sepsis and mutant homozygous individuals had increased risk of severe sepsis. Previous studies also reported that a frequent human LBP SNP (minor allelic frequency = 0.08) affecting an amino acid led to a dysfunctional LBP and had a reduced binding capacity for LPS and lipopeptides. Decreased cytokine response after LPS exposure was also identified in variant carriers. Furthermore, retrospective trial evidence suggested that this LBP SNP was correlated with increased mortality rate during sepsis and pneumonia [27]. Therefore, LBP gene polymorphisms might have an association with sepsis susceptibility.

The T → C variant in rs2232618 polymorphism leaded phenylalanine transformation leucine at amino acid 436 (Phe436Leu) in the LBP protein [28]. Therefore, rs2232618 may influence interaction for LPS and CD14. Our previous investigation reported that rs2232618C allele carriers had higher sepsis morbidity and MOD score. Mechanism research suggested rs2232618 was also related to LPS-induced activation of peripheral blood leukocytes in patients with major traumatic injury, and the rs2232618 polymorphism had impact on activities of LBP protein, but not the production of LBP protein [16]. Furthermore, Hubacek et al. showed patients which were homozygote for Phe436Leu alleles exclusively had higher mortality after sepsis [14]. Jabandziev et al. reported combing rs2232618 in LBP with additional four SNPs could be used as a predictor of sepsis outcome in children [15]. Therefore, we concluded the rs2232618 was a functional variation and might play an important role in the pathophysiologic process of sepsis and MODS. In order to further investigate the clinical association between rs2232618 and risk of sepsis in larger major traumatic patient cohorts, we enlarged the sample size in the Southwest and Southeast of China. Similar to our previous findings, individuals with more C genotype for rs2232618 polymorphism had higher incidence of sepsis in both study populations. The following meta-analysis further confirmed the association. Thus, the results presented here indicated the rs2232618 polymorphism might be a functional risk variant for sepsis in patients with major traumatic injury.

However, our study had several limitations. Firstly, owing to the lower incidence of gram-positive or mixed-infected sepsis, sub-group analysis between rs2232618 polymorphism and trauma-related sepsis was not completed. Secondly, the diagnosis criterion of sepsis had been revised as sepsis-3 for patients who had a daily SOFA score ≥ 2 with suspected infection in 2016 [29]. However, majority of our sepsis patients were diagnosed based on the sepsis-2 for patients who met ≥ 2 SIRS criteria with suspected infection, so whether the association would exist in patients identified by new sepsis criteria was unsure. Finally, we only recruited trauma patients in Chinese Han population, which is different from other ethnic populations in some aspects; further studies in other ethnic populations should be included to fully explore the association.

## Conclusions

In summary, our study enlarged the sample size to further define the clinical relation between rs2232618 and the incidence of sepsis after severe traumatic injury. The follow-up meta-analysis strongly clarified the significant association between rs2232618 and sepsis. Future studies would explore whether rs2232618 could improve early clinical therapeutic interventions in patients with sepsis.

## Abbreviations

CI: Confidence intervals
HWE: Hardy-Weinberg equilibrium
ISS: Injury Severity Score
LBP: Lipopolysaccharide-binding protein
LPS: Lipopolysaccharides
MAF: Minor allele frequency
MOD: Multiple organ dysfunction
OR: Odds ratio
SIRS: Systemic inflammatory response syndrome
SNP: Single nucleotide polymorphisms
TLR1: Toll-like receptor 1
TNF-α: Tumor necrosis factor-alpha
